# The Impact of an Incidental Dose on Axillary Tumor Control and Toxicity in Localized Breast Cancer: A Retrospective Analysis

**DOI:** 10.3390/cancers14030807

**Published:** 2022-02-04

**Authors:** Martin Schmitt, Isabelle Chambrelant, Parigna Hong Chheang, Carole Pflumio, Carole Hild, Thierry Petit, Georges Noël

**Affiliations:** 1 Radiotherapy Department, Strasbourg Europe Cancer Institute, 17 Rue Albert Calmette, 67200 Strasbourg, France; ma.schmitt@icans.eu (M.S.); i.chambrelant@icans.eu (I.C.); 2 Radiotherapy Department, Khmer Soviet Friendship Hospital, Yuthapol Khemarak Phoumin Blvd, Phnom Penh 12306, Cambodia; p.hongchheang@icans.eu; 3 Medical Oncology Department, Strasbourg Europe Cancer Institute, 17 Rue Albert Calmette, 67200 Strasbourg, France; c.pflumio@icans.eu (C.P.); t.petit@icans.eu (T.P.); 4 Breast Surgery Department, Strasbourg Europe Cancer Institute, 17 Rue Albert Calmette, 67200 Strasbourg, France; c.hild@icans.eu

**Keywords:** dosimetric analysis, breast carcinoma, radiotherapy, three-dimensional radiotherapy, intensity-modulated radiotherapy, axillary lymph node

## Abstract

**Simple Summary:**

The incidental axillary dose varies according to the whole breast irradiation technique. However, this dose has not been shown to be a prognostic factor for locoregional recurrence. The objectives of our retrospective study are to dosimetrically evaluate the incidental axillary dose according to the different irradiation techniques and the risk factors of axillary recurrence. We confirmed that the irradiation technique has an influence on the incidental dose delivered to the axillary area, but has no influence on the risk of axillary recurrence. The risk of lymphoedema could be related to the use of high tangential beams and the mean dose delivered at level II.

**Abstract:**

Purpose: The dosimetric analysis of the incidental axillary dose delivered to axillary lymph node levels I–III by different techniques of whole breast irradiation and the analysis of prognostic factors of axillary recurrence of breast cancer. Methods: We perform a retrospective analysis that includes 171 patients with localized breast carcinoma irradiated at Centre Paul Strauss. To be included in the study, patients had to have a histological confirmation of breast cancer diagnosis, surgical treatment without axillary lymph node dissection (ALND), whole breast irradiation without axillary irradiation by a specific field, and a treatment plan available. Results: Three patients had lymph node recurrence. There was no significant correlation between the maximal or mean dose delivered at the three axillary levels and the risk of axillary lymph node recurrence. There was no significant correlation between the irradiation technique and the risk of axillary lymph node recurrence. Two patients, both in the HT group, had lymphoedema. There was significantly more lymphoedema in the HT group than in the ST and IMRT groups (*p* < 0.048). The mean dose in level II was significantly higher in the group of patients with lymphoedema (3.45 Gy (1.08; 9.62) vs. 23.4 Gy (23.1; 23.6)) (*p* < 0.02). Conclusion: The irradiation technique has an influence on the incidental dose delivered to the axillary area, but has no influence on the risk of axillary recurrence. The risk of lymphoedema could be related to the use of HT and the mean dose delivered at level II.

## 1. Introduction

The management of breast cancer has evolved, particularly with regard to reducing treatment-related side effects without compromising treatment outcomes [1]. On the surgical side, the sentinel lymph node biopsy (SLNB) replaced axillary lymph node dissection (ALND) in the small tumors and in the absence of clinical lymph node involvement. This management has reduced the incidence of lymphoedema [2,3,4]. Among women with T1 or T2 invasive primary breast cancer, no palpable axillary adenopathy, and 1 or 2 sentinel metastatic lymph nodes, the ACOSOG Z0011 trial showed that omitting axillary treatment did not increase the risk of recurrence [4]. The AMAROS trial showed that the rate of lymphoedema at 5 years was lower after SLNB and radiotherapy than after ALND, 14% versus 28%, respectively [2]. To improve radiotherapy, intensity-modulated radiotherapy (IMRT) was proposed to replace three-dimensional conformal radiotherapy (3D-RT) to achieve better conformity of the target volumes and reduce unnecessary healthy tissue irradiation [5,6,7], with the perspective of reducing the side effects that negatively impact the quality of life of patients [8]. Several phase II or retrospective studies have shown a decrease in acute side effects as well as chronic breast oedema with IMRT, compared to standard 3DRT [9,10,11]. Lymphoedema is principally caused by axillary lymph node dissection (ALND) [12] and adjuvant radiotherapy, particularly when irradiation is delivered at levels I and II of the axillary area [13]. There is a positive association of lymphoedema with the increasing total dose of radiation and overlapping radiation fields [14].

In the case of whole breast or parietal irradiation, several studies have shown that a nonzero and heterogeneous dose, depending on the technique, was delivered unintentionally to the axillary area [15,16]. A study showed a trend between the irradiation technique and the risk of axillary recurrence (Schmitt et al., “A retrospective analysis of survival and prognostic factor of axillary recurrence of breast cancer”, preprint) [17]. However, to our knowledge, no study has investigated the relationship between this incident dose according to different irradiation techniques, the risk of axillary recurrence, and the risk of lymphoedema.

## 2. Methods

### 2.1. Ethical Approval

This study follows the mandatory French laws required by the CNIL (Commission Nationale de l’informatique et des libertés) and was declared to this French institution by the MR004 form, and was recorded in the HDH (Health Data Hub).

### 2.2. Patients

This monocentric retrospective analysis involved patients with localized breast carcinoma treated from 01/01/2007 to 31/12/2017 in one radiation oncology department in France, who met the following selection criteria: (i) a histologic diagnosis of invasive breast neoplasm, (ii) lumpectomy or mastectomy, (iii) whole breast irradiation/chestwall with or without irradiation of the internal mammary and/or supraclavicular areas, and (iv) dosimetry available for analysis. Adjuvant hormone therapy and (neo)adjuvant chemotherapy was allowed. The exclusion criteria were (i) ALND, (ii) breast/chestwall irradiation by electron beam, and (iii) axillary irradiation (level I and/or level II) by a specific field and (iv) metastatic disease.

Patients lay in a supine treatment position. Treatment was delivered by 3DRT or IMRT using a normofractionated or moderately hypofractionated regimen. In the case of 3DRT, radiation beams were defined as standard tangential (ST) if the beam limits were located at least 2 cm below the inferior border of the humeral head. Radiation beams less than 2 cm from the inferior border of the humeral head were defined as high tangential (HT).

One hundred sixty-three patients (95%) underwent lumpectomy, and eight patients underwent mastectomy. The median-prescribed doses at the International Commission on Radiation Units and Measurements reference point in the remnant breast, parietal wall, boost and total volume were 50.0 Gy (20.0–50.4), 50.0 Gy (46.0–50.0), 16.0 Gy (9.8–16), and 66.0 Gy (20.0–66.0), respectively. The median-prescribed fractionations were 25 fractions (5–28), 25 fractions (23–25), 8 fractions (4–8), and 33 fractions (5–33). A total of 147 patients were treated with three-dimensional radiotherapy; among them, 117 patients were treated with ST, and 30 were treated with HT. A total of 163 patients had breast or parietal irradiation without lymph node irradiation. The median breast and parietal volumes were 686.3 mL (119.0–2439.0) and 127.3.0 mL (95.0–219.6), respectively.

### 2.3. Contouring and Planning

Whole breast and parietal irradiation consisted of 3DRT or IMRT. 3DRT consisted of two opposing tangential beams. Regarding the regional node irradiation, IMNs at levels III and IV were treated with an anterior field. IMNs were treated with a combination of photons and electrons (mixed beams). IMRT consisted of rotational or nonrotational IMRT or helical tomotherapy. The clinical target volumes (CTVs) of axillary levels I–III were delineated on the basis of the European Society for Radiotherapy and Oncology (ESTRO) contouring guidelines of early-stage breast cancer [18] on Artiview software (Aquilab, Loos, France). The PTV corresponds to an isometric margin of 0.5 cm from the CTV. The same software was used to calculate the dose delivered to the three axillary levels, Ln1, Ln2, and Ln3. To enable dosimetric analysis, we performed an equivalent dose in 2 Gy per fraction

(1) $$\left(EqD2=D\times \left[d+\frac{\alpha }{\beta }]/[2+\left(\frac{\alpha }{\beta }\right)\right]\right)$$

for patients treated with hypofractionated irradiation. We chose an α/β = 4 according to the publication by Hennequin et al. [19].

### 2.4. Statistical Analysis

Categorical data were analyzed as frequency counts and percentages, whereas the measured data were evaluated using medians and ranges. Fisher’s exact test was used for the comparison of categorical variables. A Mann-Whitney test was used for the comparison of quantitative variables. The statistical analysis was carried out with R v3.6.0 software (R Core Team, Vienna, Austria).

## 3. Results

One hundred and seventy-one patients were included. The patients and treatment characteristics are summarized in Table 1 and Table 2.

The median age was 61.2 years (SD 11.6). The median follow-up was 38.5 months (2.0–123.0). The median body mass index (BMI) was 25.7 (16.0–49.4). There was significantly more regional node irradiation (IMN and supraclavicular) with IMRT (*p* = 0.021).

### Dosimetric Analysis

The volumes of Ln1, Ln2, and Ln3 and the mean and maximal doses delivered at axillary levels in Ln1, Ln2, and Ln3 are summarized in Table 1. The average volumes of Ln1, Ln2, and Ln3 were 148 mL (SD 44.2), 33.8 mL (SD 12.4), and 36.5 mL (SD 70.7), respectively. For Ln1, the median maximal and mean doses were 52.8 Gy (min–max: 15.3–69.9) and 24.5 Gy (0.6–57.8), respectively. For Ln2, the median maximal and mean doses were 30.3 Gy (0.2–61.7) and 8.05 Gy (0.1–50.5), respectively. For Ln3, the median maximal and mean doses were 12.4 Gy (0.1–53.7) and 4.45 Gy (0.0–50.7), respectively.

**Figure 1:**
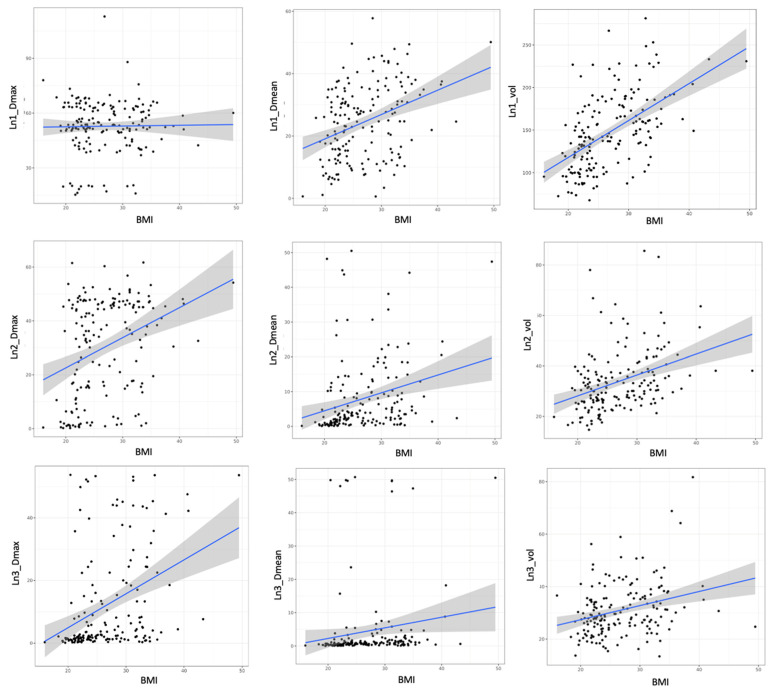
Relationship between BMI, maximal dose, mean dose, and volume of Ln1, 2, and 3. BMI: body mass index; Ln1: axillary level 1; Ln2: axillary level 2; and Ln3: axillary level 3.

**Figure 2:**
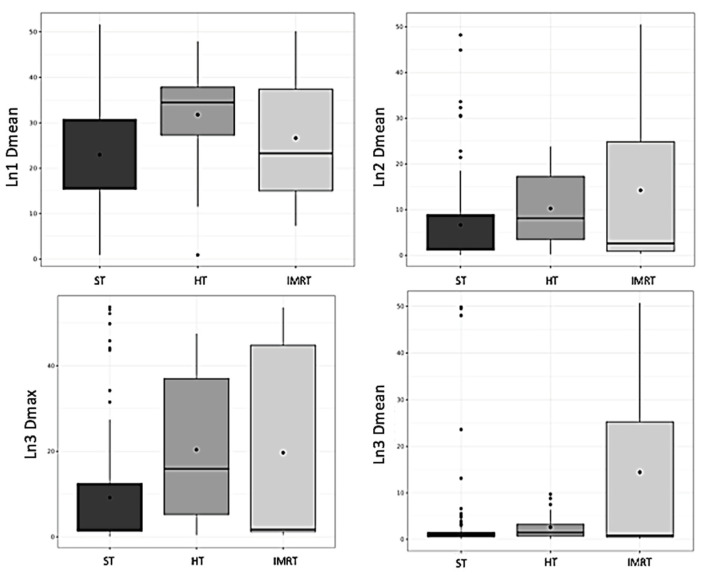
Box plot representing axillary dose according to the irradiation technique. HT: high tangential beam; Ln1: axillary level 1; Ln2: axillary level 2; Ln3: axillary level 3; and ST: standard tangential beam.

There was a significant correlation between the BMI and the volumes of Ln1, Ln2, and Ln3 (*p* < 0.001 for all) (Figure 1). There was a significant correlation between the BMI and the mean dose in Ln1, Ln2, and Ln3 (*p* < 0.001; *p* < 0.001, and *p* = 0.04, respectively). There was a significant correlation between the BMI and the maximal dose in Ln2 and Ln3 (*p* < 0.001 for both). There was a significant correlation between the BMI and irradiated breast volume (*p* < 0.001). There was a significant correlation between the targeted breast or parietal volume and the mean dose of Ln1, volume of Ln1 and Ln2, and the maximal dose of Ln2 and Ln3 (Table 3). There was a significant difference according to the irradiation technique (i.e., 3DRT vs. IMRT) concerning the mean doses of Ln1, Ln2, and Ln3, and the maximal dose of Ln3 (*p* < 0.001 and *p* = 0.016 and *p* = 0.015 and *p* < 0.001) (Figure 2). HT significantly influenced the mean doses of Ln1, Ln2, and Ln3 and the maximal doses of Ln3 (Table 4), with a significant difference according to regional node irradiation (IMN and supraclavicular area) and the mean doses of Ln1, Ln2, and Ln3 (*p* < 0.01 and *p* < 0.001 and *p* < 0.001, respectively).

Three patients developed lymph node recurrence. There was no significant correlation between the maximal or mean dose delivered at the three axillary levels and the risk of axillary lymph node recurrence. There was no significant correlation between the irradiation technique and the risk of axillary lymph node recurrence.

Two patients, both in the HT group, had grade 1 lymphoedema. There was significantly more lymphoedema in the HT group than in the ST and IMRT groups (*p* < 0.048). The dose in level II was significantly higher in the group of patients with lymphoedema than the mean dose in the group of patients without lymphoedema, 20.9 Gy and 22.8 Gy versus 3.63 Gy (1.20; 9.98), respectively, (*p* < 0.045).

## 4. Discussion

In the current study, we showed that the delivered dose to axillary levels I, II, and III varied significantly according to patient BMI and irradiation techniques. The values are consistent with several other previously published studies [15,16,20]. HT fields deliver a significantly higher mean dose at levels I, II, and III than the ST fields and IMRT. Reznik et al. were the first to compare the dosimetric impact of ST and HT fields. They showed better coverage of the axillary area by the HT field technique. The average doses delivered in levels I, II, and III with ST were 66% (SD = 13%), 44% (SD = 18%), and 31% (SD = 20%), respectively, compared to 86% (SD = 9%), 71% (SD = 19%), and 73% (SD = 17%), respectively, of the prescribed dose with HT [21].

In 2014, Belkacemi et al. retrospectively studied the dose distribution in the SLNB area visualized in 25 patients by clips. Dosimetry was calculated in 3DRT with ST and HT fields. The mean doses delivered in axillary levels I, II, and III and in the SLNB area were significantly lower with ST fields than with HT fields and were 22 Gy vs. 38 Gy (*p* = 0.004), 3 Gy vs. 11 Gy (*p* = 0.019), 2 Gy vs. 5 Gy (*p* = 0.003), and 30 Gy vs. 45 Gy (*p* = 0.02), respectively [22]. In 2016, Lee et al. described a significantly lower dose delivered in the axillary area with IMRT compared to field-in-field 3D radiotherapy (FIF-3DRT) (*p* = 0.001 for all three levels) [23].

The axillary delivered dose appears to be lower, and this difference could be explained by the degree of optimization in IMRT, the definition of HT, axillary volume, and the irradiation supraclavicular area. The definition of HT radiation fields varied among the studies. For two studies, they were defined by an upper limit of the field reaching the humeral head [21,22], and, for another, they were defined as when the upper limit of the field was less than 2 cm from the humeral head [4]. Only two studies defined the delineated axillary volume [22,24] based on the Radiation Therapy Oncology Group (RTOG) recommendations [25]. In the current study, the volume was delineated according to the ESTRO contouring guidelines [18]. Finally, in the case of supraclavicular irradiation, we showed that the dose at levels I, II, and III was higher than that in the absence of supraclavicular lymph node irradiation (Table 5). It is likely that a significant part of the dose delivered to level IV contributes to the dose delivered to the other volumes and, in particular, to level III because of the proximity of these volumes.

Borm et al. evaluated the dose delivered in levels I, II, and III according to the irradiation protocols of the AMAROS, MA-20, and ACOSOG Z0011 trials. They delineated the clinical target volumes according to ESTRO guidelines on three patients classified according to their own shape (slender, standard, and obese). The margins for the planning target volume (PTV) were not specified in the study. In the AMAROS study, the dose to the axilla was given at full patient thickness at Ln1 and Ln2 (lateral to the coracoid process) and at 3 cm depth at Ln3 [2].

The authors showed that, for HT, a similar dose distribution compared to the AMAROS treatment plan was found at axillary levels I and II. This supported earlier assumptions that irradiation may have been involved in the good results after SLND alone in the ACOSOG Z0011 trial. However, in our study, regardless of the irradiation technique and radiation scheme, the average dose delivered involuntarily at the axillary level was much lower than in the AMAROS and Z0011 trials presented in the study by Borm et al. [20] (Table 6). We are aware that it is difficult to know the exact dose received by patients in the Z0011 trial [4], but it is possible that the practical application of the results of this trial must be carried out with caution in view of the difference in the dose delivered to the axillary area when comparing the results of Borm et al. with our own [20].

We found that the risk of lymphoedema was related to the use of HT and the mean dose delivered at level II. It has been described in the literature that there is an increased risk of lymphoedema in the case of level II irradiation because it contains a higher concentration of lymph nodes [13]. The rate of lymphoedema in our study was low compared to the ACOSOG Z0011, ALMANAC, and NSABP B32 trials [4,12,26]. In the ACOSOG Z0011 trial, the one-year rate of lymphoedema in the SLND alone group was 2% [4]. In the ALMANAC trial, the 18-month rate of lymphoedema in the SLND alone group was 7% [26]. In the NSABP B32 trial, the 36-month rate of lymphoedema in the SLND alone group was 7.5% [12]. It could then be useful to delineate Ln2 to reduce the delivered dose, particularly in the context of patients with a higher risk of lymphoedema, such as those who have had ALND.

Some limitations can be disputed in this study. First this was a retrospective, single-center study with a small number of events. Therefore, it was not possible to perform a multivariate analysis. However, compared to previously published dosimetric studies, our study included more patients. The low number of events is inherent to localized breast neoplasm. The low number of events corresponding to axillary recurrence could be seen as a limitation but is comparable with the Van Wely meta-analysis [27]. Secondly, the median follow-up may seem low, but the follow-up in radiotherapy after a localized breast cancer with a favorable evolution is only 5 years in our institution, and follow-up is carried out by the gynecologists afterwards. However, in the NSABP B-04 study, the majority of the axillary relapses in the patients treated without ALND occurred within the first 2 years [28].

## 5. Conclusions

The irradiation technique has an influence on the nonvoluntary dose delivered to the axillary area, but has no influence on the risk of axillary recurrence. The average dose delivered involuntarily at the axillary level was much lower than that in the Z0011 trial. This difference may require the careful application of the findings from trial Z0011. The risk of lymphoedema could be related to the use of HT and the mean dose delivered at level II. The consideration of Ln2 as an organ at risk could be a solution for the patients most at risk of lymphoedema.

## Figures and Tables

**Table 1:** Patient characteristics and dosimetric analysis of axillary levels I, II, and III.

Characteristics	Mean (SD)	Median (Q25–75)	Min	Max	*n*
Age at diagnosis (years)	61.2 (11.6)	62.0 (52.0; 70.0)	33.0	85.0	171
Distance between the upper beam boundary and humeral head (cm)	3.12 (1.21)	3.00 (2.30; 4.03)	0.500	5.70	148
Average heart dose (Gy)	1.51 (1.87)	0.720 (0.440; 1.85)	0.120	10.2	158
Total dose (Gy)	60.9 (8.30)	66.0 (50.0; 66.0)	20.0	66.0	171
Follow-up time (month)	40.7 (20.3)	38.0 (29.0; 48.0)	2.00	123	171
Fractionation	28.8 (6.53)	33.0 (19.0; 33.0)	5.00	33.0	171
Body mass index	26.9 (5.56)	25.7 (22.4; 31.2)	16.0	49.4	170
Ki 67 (%)	19.5 (17.9)	15.0 (8.00; 21.5)	2.00	80.0	166
Number of sentinel lymph nodes removed	2.40 (1.60)	2.00 (1.00; 3.00)	0	9.00	169
Size (mm)	14.6 (7.71)	13.0 (9.50; 19.0)	1.00	45.0	163
Target breast volume	778.4 (448)	686.3 (453.6; 1040.4)	119.0	2439.0	163
Target parietal volume	135.2 (37.6)	127.3 (120.3; 139.8)	95.0	219.6	8
Ln1 vol	148 (44.2)	142 (111; 176)	67.7	281	171
Ln1 Dmax	52.8 (14.1)	53.4 (47.9; 62.8)	15.3	69.9	171
Ln1 Dmean	24.5 (12.1)	24.7 (14.2; 33.5)	0.607	57.8	171
Ln2 vol	33.8 (12.4)	31.3 (26.1; 38.0)	14.7	85.6	171
Ln2 Dmax	30.3 (18.4)	36.2 (15.0; 46.3)	0.200	61.7	171
Ln2 Dmean	8.05 (10.6)	3.70 (1.25; 10.3)	0.100	50.5	171
Ln3 vol	36.5 (70.7)	29.1 (24.8; 35.8)	13.4	946	171
Ln3 Dmax	12.4 (16.4)	3.40 (1.30; 18.5)	0.100	53.7	171
Ln3 Dmean	4.45 (11.5)	0.800 (0.405; 1.85)	0	50.7	171

Dmax: maximal dose; Dmean: mean dose; Gy: Gray; mm: millimeters; Ln1: axillary level 1; Ln2: axillary level 2; Ln3: axillary level 3; sd: standard deviation; and vol: volume of axillary level.

**Table 2:** Qualitative variables.

Characteristics		*n* (%)
Lymph node recurrence	No	165 (98%)
	Yes	3 (1.8%)
Adjuvant chemotherapy	No	136 (80%)
	Yes	35 (20%)
Neoadjuvant chemotherapy	No	162 (95%)
	Yes	9 (5.3%)
Conservative surgery	No	8 (4.7%)
	Yes	163 (95%)
Sentinel lymph node	No	2 (1.2%)
	Yes	169 (99%)
Scarff–Bloom–Richardson grade	1	64 (39%)
	2	70 (42%)
	3	31 (19%)
HER	Negative	159 (94%)
	Positive	10 (5.9%)
Histology	Invasive ductal carcinoma	155 (88%)
	Invasive lobular carcinoma	14 (8.2%)
	Medullary carcinoma	1 (0.59%)
Triple negative	No	157 (92%)
	Yes	14 (8.2%)
	Yes	150 (88%)
Laterality	Right	100 (58%)
	Left	71 (42%)
Lymphoedema	No	164 (99%)
	Yes	2 (1.2%)
Normofractionation	No	44 (26%)
	Yes	127 (74%)
Estrogen receptor positivity	Yes	153 (89%)
	No	17 (9.9%)
	NA	1 (0.58%)
Regional node irradiation	Yes	8 (4.7%)
	No	163 (95%)
Radiation technique	3DRT	117 (68%)
	HT	30 (18%)
	IMRT	24 (14%)
Standard tangential	No	30 (20%)
	Yes	119 (80%)

3DRT: three-dimensional radiotherapy; HER2: human epidermal growth factor receptor 2; IMRT: intensity-modulated radiation therapy; and NA: non-available.

**Table 3:** Influence of breast volume on the axillary dose and volume.

Variable	Correlation Coefficient (95% CI)	*n*	*p*	Test	Correlation Coefficient
Ln1 Dmax	0.0714 (−0.0795; 0.219)	171	0.35	Pearson	-
Ln1 Dmean	0.306 (0.164; 0.436)	171	<0.001	Pearson	-
Ln1 vol	0.480 (0.356; 0.588)	171	<0.001	Pearson	-
Ln2 Dmax	0.276 (0.131; 0.409)	171	<0.001	Pearson	-
Ln2 Dmean	0.117 (−0.0338; 0.262)	171	0.13	Pearson	-
Ln2 vol	0.220 (0.0720; 0.358)	171	<0.01	Pearson	-
Ln3 Dmax	-	171	<0.001	Spearman	0.363
Ln3 Dmean	−0.0713 (−0.219; 0.0796)	171	0.35	Pearson	-
Ln3 vol	0.143 (−0.00736; 0.287)	171	0.062	Pearson	-

Dmax: maximal dose; Dmean: mean dose; Ln1: axillary level 1; Ln2: axillary level 2; Ln3: axillary level 3; and vol: volume of axillary level.

**Table 4:** Influence of the tangential beam height on the axillary dose.

Variable	HT (*n* = 30)	ST (*n* = 119)	*n*	*p*
Ln1 Dmax, median	52.0 (46.8; 62.0)	53.6 (6.0; 63.2)	149	0.59
Ln1 Dmean, median	34.3 (27.0; 37.9)	23.2 (13.1; 30.2)	149	<0.001
Ln2 Dmax, median	41.9 (33.1; 47.1)	34.1 (11.8; 46.0)	149	0.066
Ln2 Dmean, median	8.10 (3.05; 14.5)	3.25 (1.00; 8.82)	149	<0.01
Ln3 Dmax, median	15.9 (3.76; 36.9)	2.40 (1.10; 12.7)	149	<0.001
Ln3 Dmean, median	1.45 (0.602; 3.00)	0.700 (0.400; 1.30)	149	<0.01

Dmax: maximal dose; Dmean: mean dose; HT: high tangential beam; Ln1: axillary level 1; Ln2: axillary level 2; Ln3: axillary level 3; and ST: standard tangential beam.

**Table 5:** Influence of supraclavicular irradiation on the axillary dose.

Variable	Supraclavicular Irradiation		*n*	*p*
	Yes (*n* = 8)	No (*n* = 163)		
Ln1 Dmean (Gy)	38.6 (30.5; 43.6)	24.6 (13.6; 32.6)	171	<0.01
Ln2 Dmean (Gy)	35.9 (28.6; 44.6)	3.30 (1.15; 9.40)	171	<0.001
Ln3 Dmean (Gy)	49.5 (47.6; 49.7)	0.700 (0.403; 1.50)	171	<0.001

Dmean: mean dose; Gy: Gray; Ln1: axillary level 1; Ln2: axillary level 2; and Ln3: axillary level 3.

**Table 6:** Comparison of the axillary dose delivered between ACOSOG Z0011, AMAROS, and our retrospective study.

Variable	AMAROS	ACOSOG		Retrospective Study	
		ST	HT	ST	HT
Ln1 Dmean (Gy)	52.6 ± 6.5	42.2 ± 13.8	48.4 ± 4.1	23.2 (3.1; 30.2)	34.3 (27.0; 37.9)
Ln2 Dmean (Gy)	49.4 ± 3.7	35.6 ± 17.6	47.5 ± 3.9	3.25 (1.00; 8.82)	8.10 (3.05; 14.5)
Ln3 Dmean (Gy)	47.3 ± 1.7	12.0 ± 12.8	44.7 ± 5.6	0.700 (0.400; 1.30)	1.45 (0.602; 3.00)

Dmean: mean dose; HT: high tangential beam; Ln1: axillary level 1; Ln2: axillary level 2; Ln3: axillary level 3; and ST: standard tangential beam.

## Data Availability

Data available on request due to privacy restrictions. The data presented in this study are available on request from the corresponding author.
